# Rethinking sources of representative controls for the conduct of case–control studies in minority populations

**DOI:** 10.1186/1471-2288-13-71

**Published:** 2013-05-31

**Authors:** Elisa V Bandera, Urmila Chandran, Gary Zirpoli, Susan E McCann, Gregory Ciupak, Christine B Ambrosone

**Affiliations:** 1 The Cancer Institute of New Jersey, Robert Wood Johnson Medical School, 195 Little Albany Street, New Brunswick, NJ, 08901, USA; 2 University of Medicine and Dentistry of New Jersey School of Public Health, Piscataway, NJ, USA; 3 Department of Cancer Prevention & Control, Roswell Park Cancer Institute, Buffalo, NY, USA

**Keywords:** African American, Case–control studies, Controls, Community controls, Minorities, Random digit dialing, Recruitment

## Abstract

**Background:**

Recruitment of controls remains a challenge in case–control studies and particularly in studies involving minority populations.

**Methods:**

We compared characteristics of controls recruited through random digit dialing (RDD) to those of community controls enrolled through churches, health events and other outreach sources among women of African ancestry (AA) participating in the Women’s Circle of Health Study, a case–control study of breast cancer. Odds ratios and 95% confidence intervals were also computed using unconditional logistic regression to evaluate the impact of including the community controls for selected variables relevant to breast cancer and for which there were significant differences in distribution between the two control groups.

**Results:**

Compared to community controls (n=347), RDD controls (n=207) had more years of education and higher income, lower body mass index, were more likely to have private insurance, and less likely to be single. While the percentage of nulliparous women in the two groups was similar, community controls tended to have more children, have their first child at a younger age, and were less likely to breastfeed their children. Dietary intake was similar in the two groups. Compared to census data, the combination of RDD and community controls seems to be more representative of the general population than RDD controls alone. Furthermore, the inclusion of the community group had little impact on the magnitude of risk estimates for most variables, while enhancing statistical power.

**Conclusions:**

Community-based recruitment was found to be an efficient and feasible method to recruit AA controls.

## Background

One of the most challenging issues in the design of case–control studies is the selection of an optimal comparison group that minimizes selection bias, while allowing cost-efficient and feasible recruitment. The overall goal of control selection is to obtain a comparison group that is representative of the source population of cases, with two basic rules: controls should be selected from the same source population where the cases come from and should be selected independently of their exposure status [1].

A commonly accepted method to select controls is random digit dialing (RDD) by which households in the target area are randomly called to identify potential eligible participants. However, new technologies such as the increasing use of cell phones, answering machines, and caller ID in people’s homes, which allows them to screen calls, have contributed to lower response rates to RDD methods [2,3]. Furthermore, area codes are no longer accurately representing area of residence [1]. Therefore, we can no longer assume that the use of RDD for control identification results in a random sample of the general population or that it will avoid selection bias in case–control studies. In fact, studies have shown that the use of RDD leads to lower participation rates [4-6] and that participants recruited through RDD tend to be more educated than the general population [4,7,8].

Despite well-documented health disparities for African Americans, their recruitment into research studies has been traditionally challenging [9]. Moorman et al. [10] encouraged the sharing of successes and pitfalls in recruiting attempts among epidemiologists to improve recruiting and perhaps to help develop alternative methods of selecting controls. In this manuscript, we describe our experience recruiting community controls and the methods used. We also compare characteristics of community controls to those identified through RDD, and discuss potential selection bias and representativeness of the two populations.

## Methods

### Study population

The Women’s Circle of Health Study (WCHS) is a multi-site case–control study in New York City (NYC) and New Jersey (NJ) aiming to evaluate risk factors for early and aggressive breast cancer in women of African (AA) and European (EA) ancestry. The study design was described in detail elsewhere [11]. In brief, cases were AA and EA women with primary, histologically confirmed invasive breast cancer or ductal carcinoma in situ, ages 20–75 years. Controls were frequency matched on age and race. Both cases and controls had to be able to understand and read English and had no previous history of cancer other than non-melanoma skin cancer.

Recruitment in NYC took place between January 2002 and December 2008 and involved hospital-based ascertainment of cases, while controls were identified through RDD, frequency matching to telephone prefixes of cases. The sampling frame was designed so that cells categorized by age were filled in similar proportions to those of the cases. Recruitment at the NJ site started in March 2006 and is ongoing. Phase I of the study (WCHS) ended in April 2012 and covered seven counties in NJ (Bergen, Essex, Hudson, Mercer, Middlesex, Passaic, and Union), but additional funding allowed us to extend the study recruitment (Phase II: WCHS2) and to include two additional counties for a total of nine counties. Analysis presented here includes only WCHS AA participants from the NJ site (recruited from March 2006 to December 2012), as community-based recruitment was not conducted in NYC or in EA participants. Cases in NJ were identified from 2006 to 2012 by the NJ State Cancer Registry using rapid case ascertainment. Controls were initially recruited though RDD (2006 to 2010) and later through community-based efforts (2009–2012) as described in more detail below. The protocol, informed consent and all study materials were approved by the Institutional Review Boards at the University of Medicine and Dentistry of New Jersey, Mount Sinai School of Medicine, and Roswell Park Cancer Institute.

### Recruitment of RDD controls

We contracted with a commercial firm to identify controls in the target areas using RDD, frequency matched to the cases by age group and race. Potentially eligible controls were then contacted by study staff to confirm eligibility and schedule an interview. However, particularly for AA women, we found that the phone numbers provided were often not useful (e.g., disconnected or wrong numbers) and many potential participants were unreachable after many attempts. Furthermore, we found RDD to be quite expensive considering the yield of controls. For example, for the period 3/1/09 – 3/31/10, $205,832 was paid to the commercial firm, during which 389 controls were enrolled; that comes to $529 in recruitment costs for each RDD control enrolled. Therefore, based on the high cost of RDD and the few controls that were being recruited, we began to consider other options. Because community-based recruitment appeared to be a successful recruiting mechanism, and the dwindling funds to conduct the study, we discontinued use of RDD for identification of controls in 2010.

### Recruitment of community controls

During the time that we were experiencing difficulties with RDD recruitment, we were receiving calls from women interested in participating in the study, as well as AA breast cancer advocates who wanted to help us recruit women for this research. Thus, we began to explore community-based recruitment in November 2009. Working closely with community representatives, AA breast cancer advocates, AA churches, senior citizen centers and cancer-related agencies (e.g., American Cancer Society), as well as breast cancer support programs, we promoted the study in the seven counties in NJ. We also sent study flyers to health providers for posting where our cases were being diagnosed (e.g., screening facilities) and also distributed flyers at numerous health fairs and cancer-related events.

Our most successful effort was recruitment through AA churches, based on the interest of the congregations. For example, in many instances, several churches in New Jersey that knew about the study approached us to invite us to present and have a recruitment event at their site. This approach was sustainable, as we received subsequent invitations, as well as continued contact from additional women who had heard about the study after each recruiting event. Our recruitment coordinator, who is a member of the AA community in NJ, reached out to churches in the different counties. Some of these churches have large memberships covering wide geographical areas. In these churches, we were usually invited to give a presentation about breast cancer and to introduce the study. After the presentation, study staff collected names and contact information of potential participants, who were called later to confirm eligibility and schedule an interview. Often, several days after the visit to the church took place, we continued to receive phone calls from women who had heard about the study from other women who attended the event and were interested in participating. We also partnered with the American Cancer Society, NJ chapter, which provided educational materials for breast cancer prevention that we handed out at these recruiting events, as well as with The Cancer Institute of New Jersey’s Office of Community Outreach, whose staff presented information about the study at various venues and represented the study at many cancer-related events. To this date, we continue to be invited back to many of these recruiting events, confirming successful partnerships with these community leaders.

Overall, the major recruitment sources have been social networks such as family, friends, and co-workers (33%), churches (20%), and health-related groups such as the American Cancer Society, clinics, wellness groups, etc. (~19%). Many controls in the ‘social networks’ category were actually indirectly recruited through the church events (e.g., it was common for friends and family members who heard about the study at a church event to refer other women to the study).

### Data collection

Standardized interviewer training and data collection methods were used for all participants. Data for the case–control study were collected through an in-person home interview during which questionnaires were administered and body measurements and a saliva sample were obtained. A self-administered food frequency questionnaire (FFQ), the Fred Hutchinson Cancer Research Center GSEL version, was also completed by the participant during the home visit with assistance from the interviewer as needed. After completing the interview, participants were given a $50 gift card.

### Statistical methods

As noted earlier, because community-based recruitment was not conducted in the NYC site or for Caucasian women, the analyses presented here are limited to AA controls in the NJ counties, including 347 community controls and 207 RDD controls. Distributions by selected socio-demographic characteristics and known or suspected risk factors for breast cancer risk were compared between community controls and RDD controls using the Chi-square test.

Eight community controls and one RDD control did not complete the FFQ and, therefore, were excluded from the analysis comparing food and nutrient intakes. Medians and means and standard deviations were computed for total calories, major nutrients and food groups in the two control groups, and distributions compared using non-parametric methods, specifically the Wilcoxon rank sum test, to calculate *P* values.

We also compared the two populations of controls to the general population by using census data. Education, marital status, and income distributions in AA women in NJ were obtained from the 2009–2011 American Community Survey (ACS) 3-year estimates provided by the US Census Bureau [12]. The ACS is a nationwide, ongoing survey that collects data on population characteristics and housing, similar to the Census, but every year, instead of every ten years, to provide inter-censal estimates of the population for the country, states, and counties [13]. Each month, the ACS is administered to a sample of housing unit addresses. ACS 3-year estimates involve 36 months of collected data, are generally provided for areas with a population of 20,000+, are considered more precise than 1-year estimates, and involve a larger sample size [14]. Response rates for ACS in NJ have remained over 93% in the past decade [15]. Since community control recruitment in our study began in 2009, the ACS 3-year estimates from 2009–2011 provide a good source of reference. Because the ACS and our study used different income categories, especially for higher incomes, we were only able to compare distributions for common income groups. Obesity prevalence was compared with 2009–2010 National Health and Nutrition Examination Survey (NHANES) rates [16].

Finally, to evaluate the impact of including the community controls, we compared the magnitude of the odds ratios including and excluding the community control group for selected variables relevant to breast cancer and for which there were significant differences in distribution between the two control groups. Odds ratios and 95% confidence intervals were computed using unconditional logistic regression and were adjusted for age and education. SAS version 9.2 (SAS Institute, Cary NC) was used for analysis.

## Results

The distributions of selected socio-demographic characteristics and known or suspected breast cancer risk factors for community and RDD controls are shown in Table 1. As expected, community controls were, on average, less educated, had lower income, more likely to be single, and have Medicaid or Medicare as a form of insurance than RDD controls. They were also slightly more likely to be uninsured, but the number of women without health insurance was small in both groups. Community controls were more likely to be obese, defined as a body mass index (BMI) greater than 30 kg/m^2^;

**Table 1:** Selected characteristics of 554 African American community and RDD controls participating in the Women’s Circle of Health Study, New Jersey, 2006-2012

	**Community (n=347)**		**RDD (n=207)**		
	**n**	**%**	**n**	**%**	***P *value**^**1**^
**Age at interview (years)**					0.32
<30	7	2.0	5	2.4	
30-39	62	17.9	44	21.3	
40-49	110	31.7	66	31.9	
50-59	103	29.7	67	32.4	
60+	65	18.7	25	12.1	
**Country of origin**					0.04
United States	312	89.9	174	84.1	
Caribbean countries	28	8.1	21	10.1	
Other	7	2.0	12	5.8	
**Marital status**					<0.05
Married or living as married	107	30.9	99	47.8	
Widowed	29	8.4	8	3.9	
Separated, divorced, or no longer living as married	63	18.2	38	18.4	
Single, never married or never lived as married	147	42.5	62	30.0	
**Highest grade of school completed**					<0.05
Less than 12^th^ grade	60	17.3	17	8.2	
High school graduate or equivalent	96	27.7	56	27.1	
Some college	98	28.2	54	26.1	
College graduate	57	16.4	53	25.6	
Post-graduate degree	36	10.4	27	13.0	
**Health insurance (multiple choices possible)**					
Medicaid	90	25.9	34	16.4	<0.05
Medicare	55	15.9	16	7.7	<0.05
Employer-provided insurance	167	48.1	133	64.3	<0.05
Pay for insurance out of pocket	11	3.2	6	2.9	0.86
I do not have health insurance	26	7.5	10	4.8	0.22
Other	36	10.4	20	9.7	0.79
**Annual income**					<0.05
Less than $15,000	89	26.5	25	12.9	
$15,000-19,999	25	7.4	14	7.2	
$20,000-24,999	26	7.7	9	4.6	
$25,000-34,999	31	9.2	19	9.8	
$35,000-49,999	46	13.7	36	18.6	
$50,000-69,999	44	13.1	31	16.0	
$70,000-89,999	31	9.2	20	10.3	
$90,000 or more	44	13.1	40	20.6	
**Body Mass Index (kg/m**^**2**^**) – Premenopausal**					<0.05
< 25	37	19.3	33	27.7	
25 – 29	51	26.6	39	32.8	
30+	104	54.2	47	39.5	
**Body Mass Index (kg/m**^**2**^**) – Postmenopausal**					0.21
< 25	15	9.7	14	15.9	
25-29	43	27.7	28	31.8	
30+	97	62.6	46	52.3	
**Age at menarche (years)**					0.38
<11	36	10.4	27	13.0	
11-12	61	17.6	30	14.5	
12-13	97	28.0	48	23.2	
13-14	69	19.9	41	19.8	
14 +	83	24.0	61	29.5	
**Parity (live births)**					<0.01
0	47	13.5	31	15	
1-2	162	46.7	122	58.9	
3-4	101	29.1	48	23.2	
≥5	37	10.7	6	2.9	
**Age at first birth (years)**					<0.05
≤ 19	138	46.2	61	34.7	
20-24	83	27.8	49	27.8	
25-30	42	14.1	32	18.2	
≥31	36	12	34	19.3	
**Breastfeeding**					<0.05
Nulliparous	47	13.5	31	15.0	
No	180	51.9	84	40.6	
Yes	120	34.6	92	44.4	
**Age at menopause (years)**					0.63
Premenopausal	192	55.7	119	57.5	
≤ 44	25	7.2	12	5.8	
45-49	45	13.0	21	10.1	
50 +	83	24.1	55	26.6	
**Ever have hormone replacement therapy?**^**2**^					0.30
Yes	25	16.2	19	21.6	
No	129	83.8	69	78.4	
**Ever have a screening mammogram?**^**3**^					0.27
Yes	252	90.6	148	93.7	
No	26	9.4	10	6.3	
**History of benign breast disease**					0.99
Yes	77	22.2	46	22.2	
No	270	77.8	161	77.8	
**Family history of breast cancer?**					0.20
Yes	39	11.2	31	15.0	
No	308	88.8	176	85.0	

^1^ Based on Chi-square test comparing community controls vs. RDD controls.

^2^ Limited to postmenopausal participants.

^3^ Limited to participants ≥ 40 years of age.

 this was particularly true for premenopausal women (54.2% vs. 39.5%, P=0.04). While the percentage of nulliparous women in the two groups was similar, community controls tended to have more children and have their first child at a younger age; they were also less likely to breastfeed their children. When we compared FFQ data from the two control groups (Table 2), we found no significant differences in food consumption between the two controls groups.

**Figure 1:**
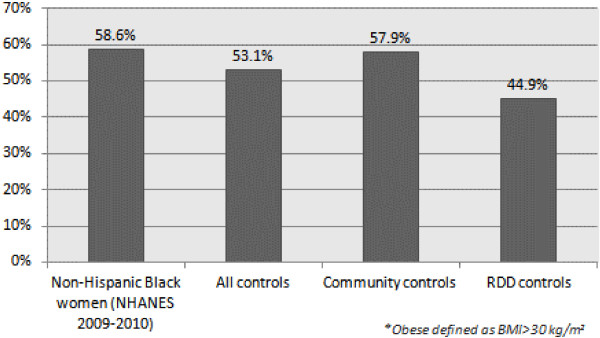
Percent obese among African American controls in WCHS vs. NHANES 2009–2010 rates (pre- and post-menopausal women combined).

Because of our concern that controls identified through RDD may have higher education and income than the general population, we compared our study population with census data to evaluate the representativeness of both control groups. As shown in Table 3, the combined community and RDD controls appeared to represent the general population better than RDD or community controls alone, at least with respect to education, income, and marital status, characteristics that were readily available in census data. When we compared the percentage of obese women in our study with that reported in NHANES for AA women (Figure 1), the same was true. Compared to NHANES data for Black women, the percentage of obese women was lower for RDD and higher for community controls, suggesting that the combination of the two control groups may be a better comparison group.

We also evaluated the potential differences in risk estimates when using only RDD controls and when including community controls as well. As shown in Table 4, risk estimates tended to be of similar magnitude including and excluding community controls for reproductive variables, and their inclusion also improved the precision of the estimates. For obesity, risk estimates were in the expected direction consistent with the literature only when including community controls.

## Discussion

Recruitment of controls remains a challenge in case–control studies. Because the goal is to obtain a comparison group that is representative of the source population of cases [1], RDD has been historically considered the best method to recruit controls in population-based studies. However, recent changes in technology have made this method less efficient and concerns have been raised about potential biases introduced by RDD sampling [6]. Minorities are underrepresented in research studies in general, but there is particularly a special need for more data on cancer in

**Table 2:** Consumption of selected food groups and nutrients in African American community and RDD controls participating in the Women’s Circle of Health Study, New Jersey, 2006-2012

	**Community (n=339)**		**RDD (n=206)**		
	**Median**	**Mean (SD)**	**Median**	**Mean (SD)**	*P *value^*^
Calories (kcal/day)	1606.0	1794.6 (998.0)	1569.4	1845.1 (1261.9)	0.61
Total fat (g/day)	56.9	66.9 (41.8)	54.2	68.4 (52.6)	0.41
Total protein (g/day)	64.3	71.5 (39.1)	65.7	73.3 (49.3)	0.54
Total carbohydrates (g/day)	206.2	231.6 (134.7)	198.1	234.9 (163.89)	0.46
Total dietary fiber (g/day)	15.2	16.8 (9.1)	15.1	16.3 (9.4)	0.33
Beta-carotene (mcg/day)	3248.5	4239.7 (3247.3)	3364.2	4178.9 (3156.6)	0.91
Vitamin C (mg/day)	88.8	111.2 (108.4)	84.9	122.4 (132.3)	0.63
Vitamin D (mcg/day)	3.7	4.93 (4.2)	3.7	5.0 (4.7)	0.73
Vitamin E (IU)	11.5	14.2 (10.6)	11.1	15.7 (19)	0.43
Folate (mcg/day)	365.6	391.1 (220.4)	355.2	398.5 (260.7)	0.59
Calcium (mg/day)	697.6	811.5 (561.5)	657.2	807.5 (594.7)	0.42
Total dairy (g/day)	169.2	229.5 (241.3)	135.0	210.3 (226.0)	0.13
Total vegetables (g/day)	160.2	201.0 (170.4)	177.1	215.1 (167.2)	0.29
Total fruit (g/day)	157.2	231.2 (256.5)	139.3	207.4 (201.8)	0.33
Total meat (g/day)	38.3	54.2 (62.3)	38.2	58.1 (61.0)	0.60

*P values based on Wilcoxon rank sum test.

**Table 3:** Distribution of selected characteristics for RDD, community controls, all controls, and census estimates

	**RDD controls (%)**	**Community controls (%)**	**All controls (%)**	ACS* (%)
**Education**				
Less than 12^th^ grade	8.2	17.3	13.9	14.6
High school	27.1	27.7	27.4	31.8
Some college	26.1	28.2	27.4	31.2
College graduate	25.6	16.4	19.9	22.4^†^
Post-graduate degree	13	10.4	11.4	
**Annual income**				
Less than $15,000	12.9	26.5	21.5	18.1
$15,000-19,999	7.2	7.4	7.4	5.4
$20,000-24,999	4.6	7.7	6.6	5.1
$25,000-34,999	9.8	9.2	9.4	10.9
$35,000-49,999	18.6	13.7	15.5	13.2
**Marital status**				
Married	46.8	29.9	36.2	26.8
Widowed	3.9	8.5	6.8	9.3
Separated	5.4	5	5.2	4.3
Divorced	13.3	13.5	13.4	10.8
Single	30.5	43	38.4	48.8

*2009-2011 American Community Survey 3-year estimates for New Jersey African American Female; US Census Bureau. ^†^percentage represents both college graduate and post-graduate degree

 minority populations [9]. This is particularly true for breast cancer, for which evidence in AA women is limited [17,18]. Therefore, exploring new methods to improve minority recruitment, and for control selection, is particularly important in breast cancer studies of AA women.

Because community recruitment uses non-random methods to identify controls, we were concerned that the use of this control group may introduce selection bias. We compared socio-demographic, reproductive, and lifestyle factors between controls recruited through RDD and community-based efforts, and found differences in some of these factors. For example, we found that RDD controls had higher socioeconomic status (higher education and income levels), which is consistent with what has been reported by others [7,8]. Importantly, education, income, and marital status distributions among the community controls and among the combined RDD and community controls mirrored the general population distributions for AA women according to NJ census data more closely than RDD controls alone. Furthermore, comparing case and control characteristics, the addition of the community controls did not substantially affect risk estimates, while providing more statistical power. The fact that the group of combined controls tended to be more representative of the source population lends more confidence to the estimates computed including the community controls. To further assess potential selection bias in analyses of data, we routinely compare results including and excluding community controls. If we were to find differences in estimates, we would present results both including and excluding community controls so that the reader could draw their own conclusions. Similar to the results we presented in this manuscript, results do tend to be in the same direction, while estimates are more precise given the larger population when including the community controls.

As noted earlier, one of the rules for an appropriate comparison group in case–control studies is that the controls are selected from the same source population as the cases [1]. We were reassured that the population that we were covering with RDD recruitment, case recruitment, and community-based recruitment came from the same source population when we received several calls from RDD controls who had actively or passively refused

**Table 4:** Comparison of selected results including and excluding community controls, Women’s Circle of Health Study, New Jersey, 2006-2012

	**Cases vs. all controls**		**Excluding community controls**	
	**OR**^**1**^	**95% CI**	**OR**^**1**^	**95% CI**
Breastfeeding in parous women (yes vs. no)	0.88	0.66-1.18	0.73	0.49-1.07
Parity (>3 live births vs. none)	0.79	0.52-1.18	0.94	0.54-1.64
Age at first birth (>30 vs ≤30)	0.75	0.49-1.15	0.66	0.39-1.12
Obesity (BMI≥30 vs. BMI<25) [premenopausal women only]	0.86	0.54-1.37	1.17	0.64-2.14

^**1**^Adjusted for age and education.

OR: odds ratio; CI: confidence interval; BMI: Body Mass Index in kg/m^2^.

 and later changed their mind and wanted to participate in the study after seeing it advertised in their community, as well as from cases who refused earlier or were just diagnosed and contacted us to participate even before the NJ State Cancer Registry received their diagnoses. Another rule is that controls are selected independently of their exposure status [1], which should not be a concern in our community-based efforts, as all women meeting our study criteria were invited to participate and those criteria were unrelated to the exposures of interest.

Although investigators are able to calculate response rates for cases if the study is population based, or from hospitals where all pathology reports are available to identify eligible cases, finding a true denominator for the majority of eligible controls is very difficult. For RDD, the numbers of those who are called through RDD, say that they fit the eligibility criteria, and agree or do not agree to be contacted by the interviewer or study staff can be calculated. But does this really represent the true response rate? It is likely that a proportion of households do not answer the telephone when it is a number they do not recognize, or if they do answer, do not answer truthfully if there is a person in the household who meets the eligibility criteria. Thus, the ‘response’ rate is likely misleading. One also cannot document response rates from community participants because the true denominator is unknown. Perhaps it is time to reconsider issues of response rates for controls in this era when there is much less openness to unsolicited telephone calls, or willingness to participate in research studies. Particularly for minority participants, the best approach may be community events where there can be education and outreach, with attention to the populations from which these groups arise, and if they are likely to represent the same population as cases.

The problems with finding the ideal control group may appear to make case–control studies less attractive, with attention turning to cohort studies. However, for studies of rare cancers, or in special populations, case–control studies may be the best way to find answers to pressing questions. More importantly, case–control studies may allow for more in-depth translational research, with capabilities to rapidly identify cases, and to obtain tumor tissue blocks and medical record and treatment information in an easier, more timely fashion. As we learn more and more about the heterogeneity of various cancers, having the ability to examine tumor tissues for molecular characteristics will allow for better classification of disease and therefore, clearer associations regarding etiologic factors.

There are limited data evaluating alternative methods to recruit minorities in epidemiologic studies. In agreement with our study, Cabral et al. [19] found RDD to be extremely labor intensive and not efficient to recruit AA and Latino controls in a case–control study of lung cancer, and explored community-based methods, similar to ours. Similar to our experience, they found the community-based control group to be more representative of the San Francisco Bay Area AA population than the RDD control group or the HCFA (Health Care Financing Administration) control group which was limited to controls older than 65 years.

Several barriers to minority participation in research studies have been identified including historical mistrust, economic, socio-cultural, knowledge and accessibility issues [9]. Strategies that have been proposed to increase minority recruitment in research studies include involving members of the target population in recruiting efforts [9,20], addressing misperceptions that potential participants may have about research [21], using face-to-face outreach and recruitment methods [21,22], offering monetary incentives, showing respect for individuals, appreciating cultural differences, and “giving back” to the community [23]. We adopted all these strategies in our community-based recruitment efforts, and our approach proved to be better for recruiting minority populations than RDD or other methods. Community-based recruitment was also cost-efficient, as it allowed us to use our existing resources and personnel, rather than paying an external firm to identify RDD controls. Moreover, potential participants approached us with interest in participating (rather than some commercial firm contacting them about the study), due to which they were much more likely to actually do the interview.

## Conclusions

In our experience, community-based recruitment was an efficient and feasible method to recruit minorities, especially when combined with RDD recruitment. However, with the growing issues regarding success of telephone contact for participation in studies, the utility of RDD will need to be monitored going forward, and may prove too expensive and less efficient for optimal recruitment of controls. Although successful for us, particularly in the AA community, where churches and community groups tend to be important, we are aware that community-based recruitment may not work in certain populations (for example, in rural underdeveloped areas) or in multi-site studies where standardization of community strategies would not be possible. Therefore, recruitment strategies should be tailored to the target population, while taking into account resources.

In our study, community-based recruitment helped us stay in touch and “give back” to the community from which we were recruiting, while supplementing the RDD controls to achieve a comparison group more representative of the general target population. We learned that for community efforts to achieve an unbiased comparison group, strategies have to be tailored to the community being recruited. Therefore, partnering with community agencies, cancer support groups, community members interested in the topic, and particularly with churches and church leaders, is crucial for the efforts to be successful. Furthermore, recruiting efforts should occur in several fronts (churches, health events, clinics) so that different demographics are represented, and in a variety of locations representing the target population. Importantly, characteristics of the participants and responses to interview data should be closely monitored in ‘real time’, so that the appropriateness of the controls in relation to cases in the study can be frequently appraised and adjusted, if necessary. From our experience, however, community-based recruitment appears to be a cost-effective method to recruit AA controls in case–control studies.

## Abbreviations

AA: African ancestry; CI: Confidence interval; EA: European ancestry; NJ: New Jersey; NYC: New York City; OR: Odds ratio; RDD: Random digit dialing; WCHS: Women’s Circle of Health Study

## Competing interests

The authors declare that they have no competing interest.

## Authors’ contributions

EB and CA designed the study. EB and CA and GC directed its implementation. EB and UC designed the analytic strategy. UC obtained NJ Census data. UC and GZ conducted statistical analyses. SM provided expertise in the interpretation of results. EB wrote the first draft of the manuscript and all co-authors provided substantive comments and editorial review. All authors read and approved the final manuscript.

## Pre-publication history

The pre-publication history for this paper can be accessed here:

http://www.biomedcentral.com/1471-2288/13/71/prepub
